# A predation cost to bold fish in the wild

**DOI:** 10.1038/s41598-017-01270-w

**Published:** 2017-04-27

**Authors:** Kaj Hulthén, Ben B. Chapman, P. Anders Nilsson, Lars-Anders Hansson, Christian Skov, Jakob Brodersen, Jerker Vinterstare, Christer Brönmark

**Affiliations:** 1 Department of Biology - Aquatic Ecology, Ecology Building, Lund University, SE-223 62 Lund, Sweden; 2 Division of Evolution and Genomics, School of Biological Sciences, University of Manchester, Oxford Rd, Manchester M139PL UK; 3 Department of Environmental and Life Sciences - Biology, Karlstad University, SE-651 88 Karlstad, Sweden; 4 National Institute of Aquatic Resources, Technical University of Denmark (DTU), Vejlsøvej 39, Silkeborg, 8600 Denmark; 5 Department of Fish Ecology and Evolution, EAWAG Swiss Federal Institute of Aquatic Science and Technology, Center for Ecology, Evolution and Biogeochemistry, Seestrasse 79, 6047 Kastanienbaum, Switzerland

## Abstract

Studies of predator-mediated selection on behaviour are critical for our understanding of the evolution and maintenance of behavioural diversity in natural populations. Consistent individual differences in prey behaviour, especially in the propensity to take risks (“boldness”), are widespread in the animal kingdom. Theory predicts that individual behavioural types differ in a cost-benefit trade-off where bolder individuals benefit from greater access to resources while paying higher predation-risk costs. However, explicitly linking predation events to individual behaviour under natural conditions is challenging and there is currently little data from the wild. We assayed individual behaviour and electronically tagged hundreds of fish (roach, *Rutilus rutilus*) before releasing them into their lake of origin, thereby exposing them to predation risk from avian apex predators (cormorants, *Phalacrocorax carbo*). Scanning for regurgitated tags at the cormorant roosting site provided data on individual predation events. We found that fish with higher boldness have a greater susceptibility to cormorant predation compared to relatively shy, risk-averse individuals. Our findings hereby provide unique and direct evidence of behavioural type-dependent predation vulnerability in the wild, i.e. that there is a predation cost to boldness, which is critical for our understanding of the evolution and maintenance of behavioural diversity in natural populations.

## Introduction

Most animals in nature constitute potential prey and studies of predator-mediated selection on phenotypic traits are critical for our understanding of the evolution and maintenance of phenotypic diversity in natural populations. In the few empirical studies of predator-mediated selection in natural populations, morphological traits are almost exclusively the focus, as it is sometimes possible to ascertain the size and shape of the morphological characters of prey following a predation event, such as horn size in the flat-tailed horned lizard *Phyrnosoma mcalli* ^1^ or wing shape in the damselfly *Calopteryx splendens* ^2^. Other aspects of the phenotype, such as an individual’s behaviour, are extremely difficult to relate to predation vulnerability in the wild, as behaviour cannot be readily quantified following a predation event.

A recent focus on individual variation in animal behaviour has revealed that consistent intraspecific individual differences in behaviour is a taxonomically ubiquitous phenomenon documented across a dazzling array of animals, from ants to fish, birds and primates^3–5^. While the existence of between-individual variation in behavioural traits such as boldness, i.e. the propensity to take risks^6^, is no longer contentious, empirical studies investigating the ecological forces that maintain such variation, particularly under natural conditions in the wild, remain scarce^7, 8^. A central axiom of the debates surrounding the maintenance of behavioural variation in wild populations is that individual behavioural types differ in a cost-benefit trade-off where risk-prone, bold individuals may access greater rewards, including resources and mates, but at the cost of exposure to higher risks, such as an increased probability of predation^9–11^. Predation risk has hereby emerged as a likely selection pressure behind the evolution of individual variation in risk-taking, potentially acting as an ecological mechanism to balance the costs and benefits of different behavioural strategies in the wild^10, 12, 13^.

However, observing and quantifying predation events on individual animals with a known behavioural phenotype under natural conditions is extremely challenging, but absolutely essential in order to obtain direct evidence for behaviour-dependent predation risk^7^. A growing body of circumstantial evidence supports the idea that certain behavioural types experience elevated predation vulnerability. For example, a correlatory multi-lake study linked patterns of habitat use in domestic and wild strains of rainbow trout (*Oncorhynchus mykiss*) stocked into multiple experimental lakes with their recapture rate, a proxy for survival in this study. Wild trout, that used less risky habitat, had a higher recapture rate than domesticated trout^11^. In contrast, analysis of a long-term dataset documenting the survival of bighorn sheep ewes *Ovis canadensis* is suggestive of moderate selection acting to favour bold ewes^14^. Whilst both studies revealed a link between risk-taking behaviour and survival, neither study could reveal the agents responsible for the observed pattern of survival, which is understandable given the logistical challenges involved in such an endeavour. Here we address this lacuna by combining laboratory characterisation of individual risk-taking phenotypes with a method of retrieving explicit records of individual predation events in the wild, which allow us to directly test if individual boldness can predict predation risk.

We followed the fate of a large number of behaviourally typed individuals of a freshwater fish, roach *Rutilus rutilus*, under predation risk from cormorants (*Phalacrocorax carbo* spp), a key avian predator^15^. Roach were captured by electrofishing in a shallow lake in southern Sweden during autumn in two consecutive years (2009 and 2010) and brought to the lab where we quantified latency to emerge from a safe refuge as an index of individual risk-taking propensity, a repeatable personality trait in this population^16^. Following behavioural assessments we implanted individually coded electronic tags in all fish before releasing them back into the wild. Cormorants prey upon tagged fish and regurgitate indigestible tags at a well-defined roost in the lake (Supplementary Information, Fig. 1A,B). After allowing for several years of natural predation in the wild we used portable tag detectors to search the roost and retrieve the unique identity codes from cormorant-killed fish. Our data support the prediction that bold individuals pay a greater predation cost than shy individuals, which to our knowledge provides the first direct evidence of a predation cost to boldness from a wild population of animals.

## Results and Discussion

**Figure 1:**
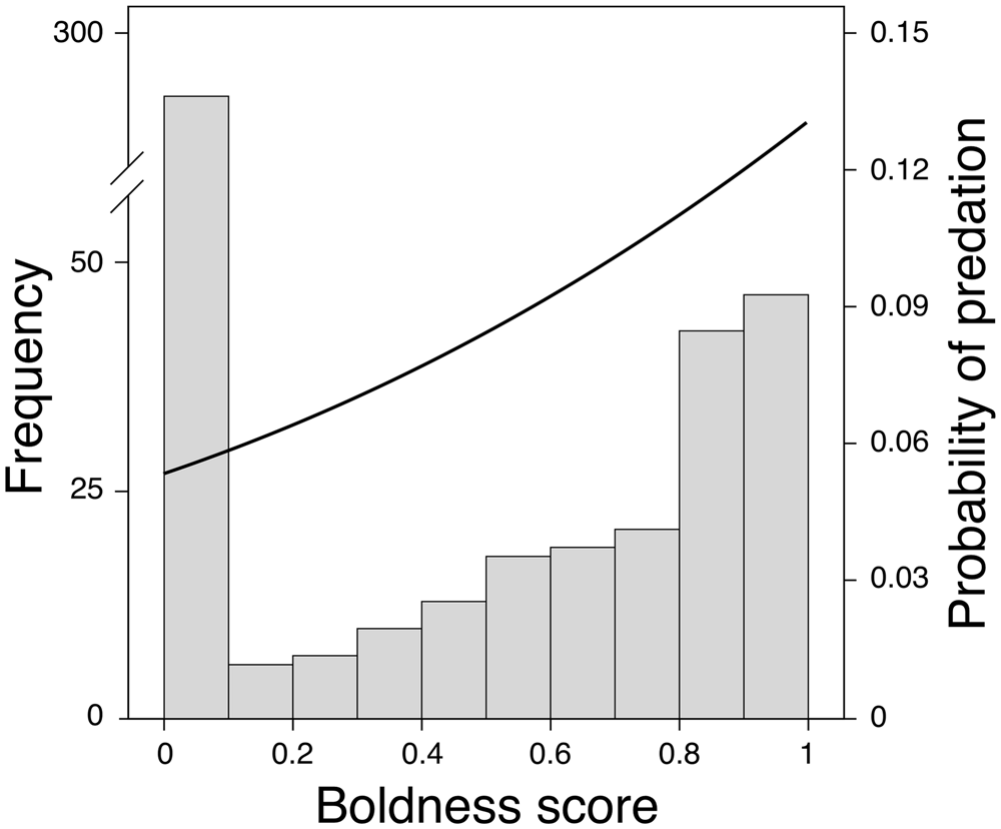
Frequency distribution (bars, left y-axis) of boldness scores overlaid with the boldness-dependent probability of cormorant predation (curve, right y-axis). Fish that had not left the refuge at 1200 s were given a boldness score of 0.

In total, we recovered 35 known individual predation events. As predicted, the probability of cormorant predation increased with boldness score (Fig. 1; Wald = 4.597; p = 0.032), whereas neither fish body size nor the body size × boldness interaction term affected the probability of predation (Wald ≤ 2.691; p ≥ 0.101). Bolder individuals thus have a significantly higher risk of being predated by cormorants, and therefore pay a greater predation cost than shy individuals.

Our results demonstrate how predators can act as agents of selection on behaviour in prey, and explicitly link a widely distributed and ecologically important behavioural trait, boldness, with susceptibility to predation in the wild. This causality from natural settings is in line with previous laboratory experiments^7, 10^ and correlative data from field studies^17, 18^, but here we unambiguously show a cost to boldness directly in nature, which has hitherto proven elusive, but is critical for the understanding of the evolution and maintenance of behavioural diversity in natural populations. The precise mechanisms behind boldness-dependent predation vulnerability warrant further attention, but could include an increased encounter rate due to the commonly reported correlation between boldness and activity^16^, a reduced social tendency and/or an increased probability of positioning at the front of a social group^19^, all of which can increase individual risk^20^.

Many of the world’s apex predators have suffered serious declines due to anthropogenically induced environmental changes^21, 22^. Cormorants were hunted to near extinction in Europe during the 19^th^ century, but as a response to successful conservation incentives have shown tremendous range recovery and a 30-fold population density increase over the last three decades^23, 24^, rapidly changing the landscape of predation risk for prey phenotypes in nature. The increased cormorant piscivory conveys potential effects on fish community composition, biodiversity and recreational values^25^, while cormorant selectivity for bolder roach may influence also the population structure of roach in the context of behavioural variation with potential repercussions for lake ecosystem dynamics and function^26, 27^.

## Methods

### Study site

The study was conducted in Lake Kranksjön situated in southern Sweden (55°42′N, 13°28′E), approximately 20 km east of the city of Lund. The lake has a surface area of 3.4 km^2^, a mean depth of 0.7 m, a maximum depth of 3.0 m, is macrophyte rich (mainly Charophytes), and moderately eutrophic^28, 29^. Gillnet surveys have shown that roach (*Rutilus rutilus*) numerically dominate the fish assemblage in the lake. Other common species are perch (*Perca fluviatus*), rudd (*Scardinius erythrophtalmus*), tench (*Tinca tinca*), pike (*Esox lucius*), bream (*Abramis brama*), and white bream (*Blicca bjoerkna*). Cormorants were counted on 54 to 119 occasions per year between 2009 and 2013, i.e. when the released fish were exposed to cormorant predation, and mean numbers of cormorants observed per day on the lake ranged between 21 and 59.

### Fish capture and boldness assay

We caught roach by electrofishing in the littoral zone of the lake in September 2009 and 2010. Captured individuals (2009: n = 294; mean total length = 136.7 mm, s.e. = 0.5, range = 122–165 mm, and 2010: n = 166; mean total length = 154.8 mm, s.e. = 1.5, range = 124–231 mm) were directly transported to experimental facilities at Lund University (55°42′ N, 13°12′ E) and acclimatized in large, opaque polyethylene containers for one week before participating in behavioural assays. Next, we measured the time taken to emerge from a refuge box, a standard protocol to score individual boldness^17^. Fish were introduced to a refuge box (28 × 20 × 20 cm) that had a remotely controlled door and was made of grey PVC. The refuge box was placed within a novel arena and to decrease environmental disturbance, a tarpaulin tent sheltered the experimental set-up. Each trial started when one individual in the holding tanks was haphazardly chosen and transferred to the refuge box. Following a 30 min acclimatization period, we lifted the door and quantified the time for fish to emerge completely from the refuge box. This time was used to calculate an index of boldness (see below), with a ceiling value of 1200 s if the fish had not left the refuge box after 20 min. In order to minimize fish handling and laboratory holding time, and thus stress of the study subjects, behavioral test was only trialed once with individual fish, as pilot work showed that refuge emergence propensity is a highly consistent trait in this population (r = 0.72 ± 0.06, F = 6.08, p = 0.015).

### Fish tagging and recovery

Following the behavioural assay, we measured the total length (TL) and tagged all fish according to^30^, by surgically implanting individually coded TIRIS Passive Integrated Transponder (PIT) tag (Texas Instruments, RI-TRP-RRHP, Plano, Texas, USA, half duplex, 134 kHz, 23.1 mm long, 3.85 mm diameter, 0.6 g in air) into the coelomic cavity of the fish. This method of PIT tagging has no significant effect on survival or body condition in roach^30, 31^. We then transported all fish back to the lake of origin where they were released at the approximate location of capture. Cormorants prey upon tagged fish and regurgitate indigestible tags at their roosting site. Between April and December 2013 (i.e. 2.5–3.5 years after the fish were released back into the lake) we performed extensive scans for PIT-tags at the only cormorant roosting site of the lake (Supplementary Information; Fig. 1A,B). An operator used a battery-powered and portable high-performance HDX backpack reader (Oregon RFID, Portland, Oregon) with an attached antenna pole (length 1.85 m, diameter 0.5 m). Once within the electromagnetic field generated by the antenna (read range: *c*. 74 cm) the PIT-tag is energized and transmits a unique alphanumeric identity code that is stored on the data logger. We performed extensive scannings of the whole roosting area on 9 occasions between 8 April and 18 December 2013. Tag data recovery was achieved by systematically sweeping with the antenna pole over the whole area. Since branches from the trees overhang the shallow water surrounding the roosting site, scanning also included the lake bottom 5 meters from shore. On average, each individual tag was detected at 5.8 of in total 9 scanning occasions.

All experimental protocols in this study were evaluated and approved by Malmö/Lund authority for ethics of animal experimentation (licence M36-14) and the used methods were in accordance with relevant guidelines and regulations.

### Data treatment and statistical analysis

Tag recovery data enabled us to link predation events in the wild to specific individuals with a known boldness score and this allowed us to relate predation risk to the degree of individual boldness. Since behavioural assays and tagging followed identical protocols during 2009 and 2010, and tag recovery occurred more than two and a half years after the last fish was released back into the lake, we pooled all individuals for our statistical analysis. To convert individual refuge emergence times to boldness scores (i.e. to make high scores indicate bold individuals) we applied the formula:

$$B=1-(t/1200)$$

where *B* is boldness score (range 0–1) and *t* is the latency (seconds) for an individual to emerge from the refuge in the behavioural assay. A logistic regression model (likelihood ratio backwards elimination with selection criteria at α = 0.05) was fitted to model the binary outcome of known predation (yes/no) as a function of the independent factors individual boldness score, body size at tagging, as well as their interaction term. Statistical analyses were performed using SPSS (SPSS Inc., Chicago, IL, USA), version. 23.0. Data available from the Dryad Digital Repository: http://dx.doi.org/10.5061/dryad.1k7h7

## Electronic supplementary material

Cormorant roost and PIT-tag
