# Supplementary material for: A predation cost to bold fish in the wild
Source: Sci Rep. 2017 Apr 27;7:1239. doi: 10.1038/s41598-017-01270-w (PMC5430796; doi:10.1038/s41598-017-01270-w)
Supplement: Supplementary file 1 — Cormorant roost and PIT-tag [file 41598_2017_1270_MOESM1_ESM.pdf]

Supplementary material to the manuscript:

A predation cost to bold fish in the wild

Kaj Hulthén[1*†], Ben B. Chapman[2†], P. Anders Nilsson[1, 3], Lars-Anders Hansson[1], Christian Skov[4], Jakob Brodersen[5], Jerker Vinterstare[1] and Christer Brönmark[1]

[1]Department of Biology - Aquatic Ecology, Ecology Building, Lund University, SE-223 62 Lund, Sweden.

[2]School of Biological Sciences, Division of Evolution and Genomics, University of Manchester, Oxford Rd, Manchester M139PL, UK.

[3]Department of Environmental and Life Sciences - Biology, Karlstad University, SE-651 88, Karlstad, Sweden.

[4]National Institute of Aquatic Resources, Technical University of Denmark (DTU), Vejlsøvej 39, Silkeborg 8600, Denmark.

[5]Department of Fish Ecology and Evolution, EAWAG Swiss Federal Institute of Aquatic Science and Technology, Center for Ecology, Evolution and Biogeochemistry, Seestrasse 79, 6047, Kastanienbaum, Switzerland.

*Corresponding author: Kaj Hulthén mail: kaj.hulthen@biol.lu.se; Tel.: +46462228437; Address: Department of Biology, Lund University, Ecology Building, SE-223 62 Lund, Sweden.

† K. Hulthén and B.B. Chapman contributed equally to this work

SUPPLEMENTARY INFORMATION

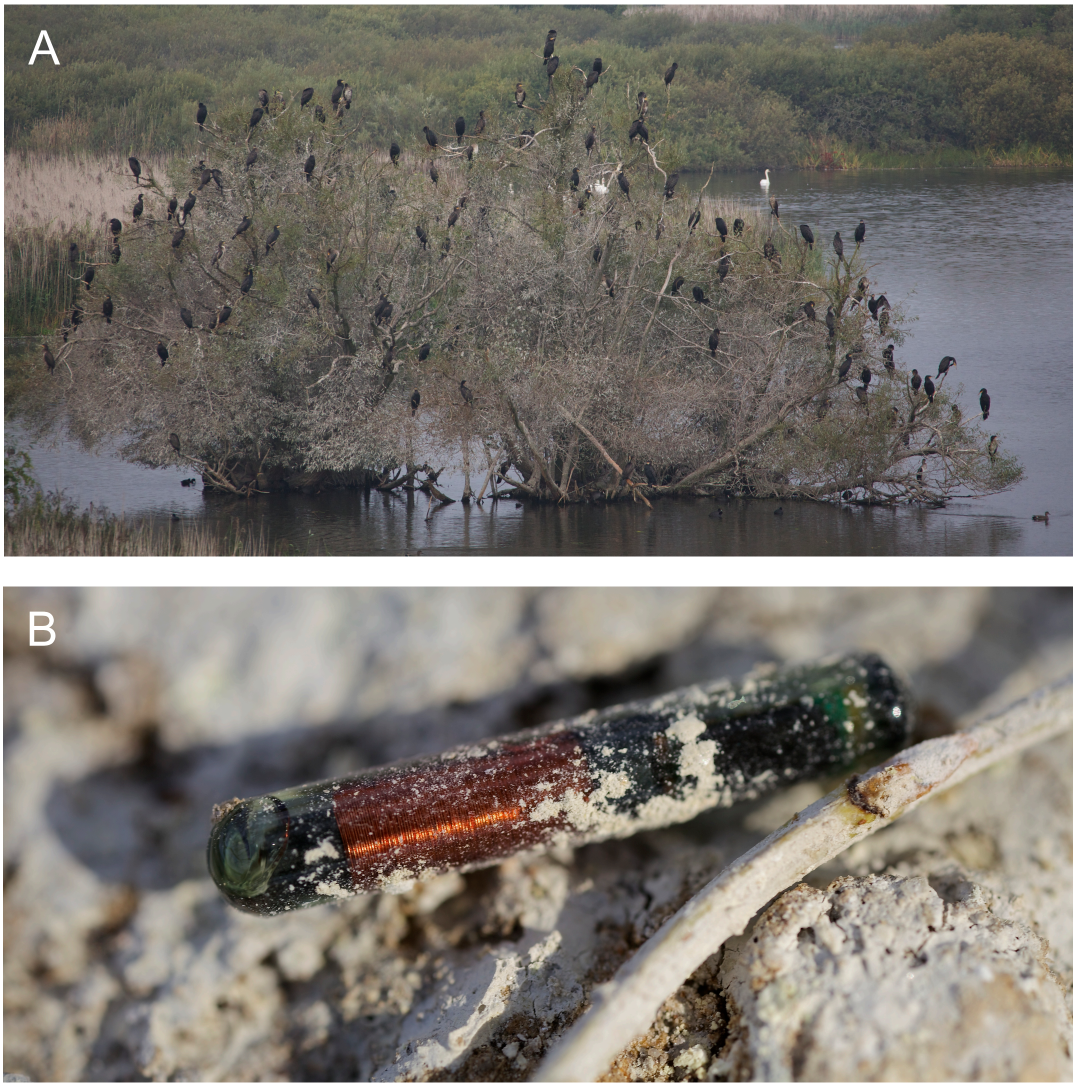

Figure 1 (A) Cormorants congregate to roost in lake Krankesjön after a day of fishing [Photo: Kaj Hulthén] (B) Electronic PIT tags with unique identity codes, originally implanted in behaviourally assayed roach and subsequently consumed by cormorants, are regurgitated at the well-defined roost [Photo: Jerker Vinterstare].
